# Supplementary material for: Omicron Spike confers enhanced infectivity and interferon resistance to SARS-CoV-2 in human nasal tissue
Source: Nat Commun. 2024 Jan 30;15:889. doi: 10.1038/s41467-024-45075-8 (PMC10828397; doi:10.1038/s41467-024-45075-8)
Supplement: Supplementary file 1 — Supplemental Information [file 41467_2024_45075_MOESM1_ESM.pdf]

SUPPLEMENTAL INFORMATION

Omicron Spike confers enhanced infectivity and interferon resistance to SARS-CoV-2 in human nasal tissue

Shi, Li et al

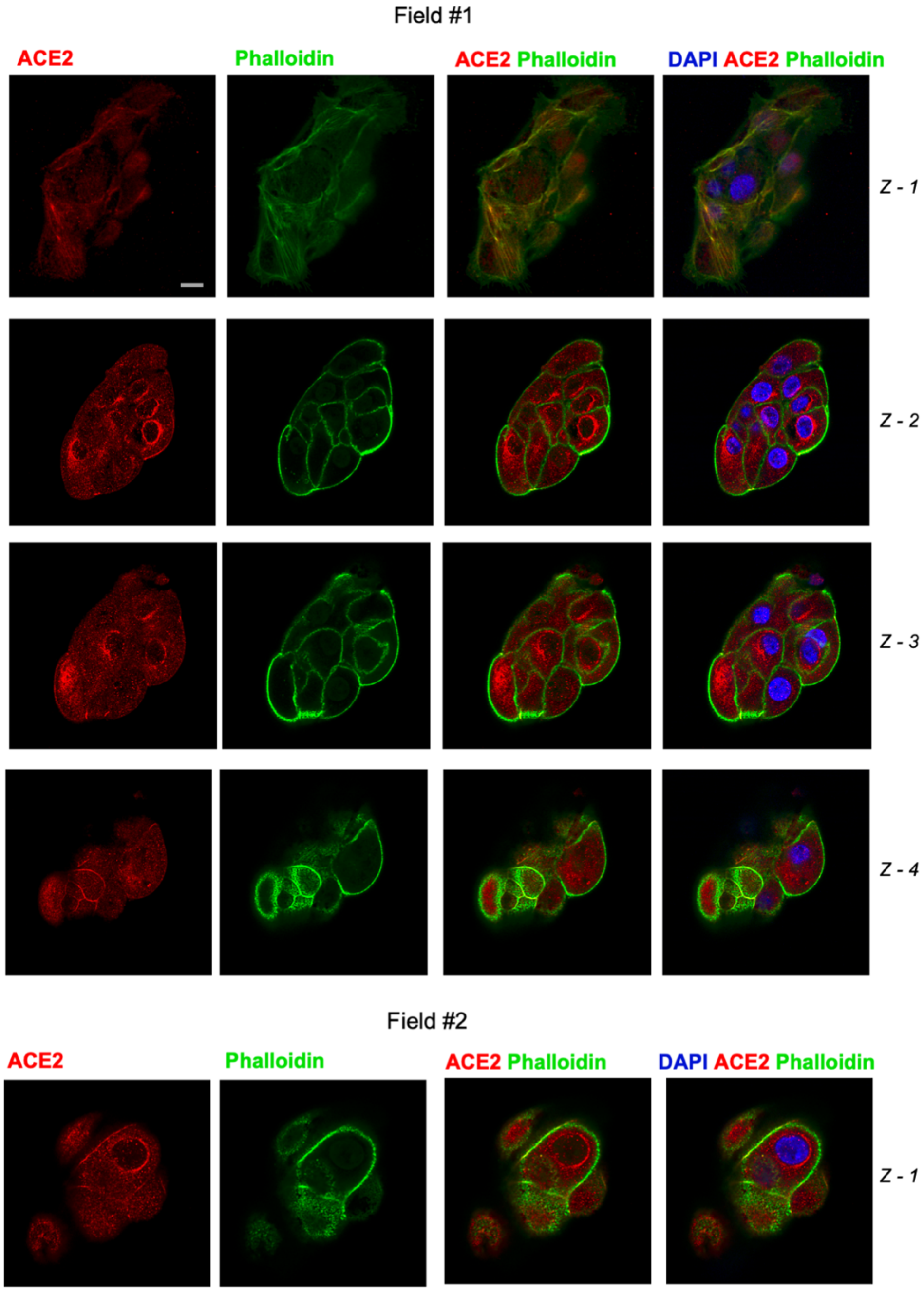

Supplemental Figure 1. Measurement of human ACE2 protein levels in submerged nasal monolayers by confocal immunofluorescence microscopy.

Primary human nasal epithelial cells (pooled from 3 human donors) were cultured as undifferentiated, submerged monolayers. Cells were fixed and permeabilized for confocal immunofluorescence microscopy. ACE2 levels were measured by anti-ACE2 immunofluorescence, the cell surface was visualized with phalloidin, and DAPI was used to stain nuclei. Confocal images from single Z slices from two separate fields are shown. Scale bar = 10 microns.

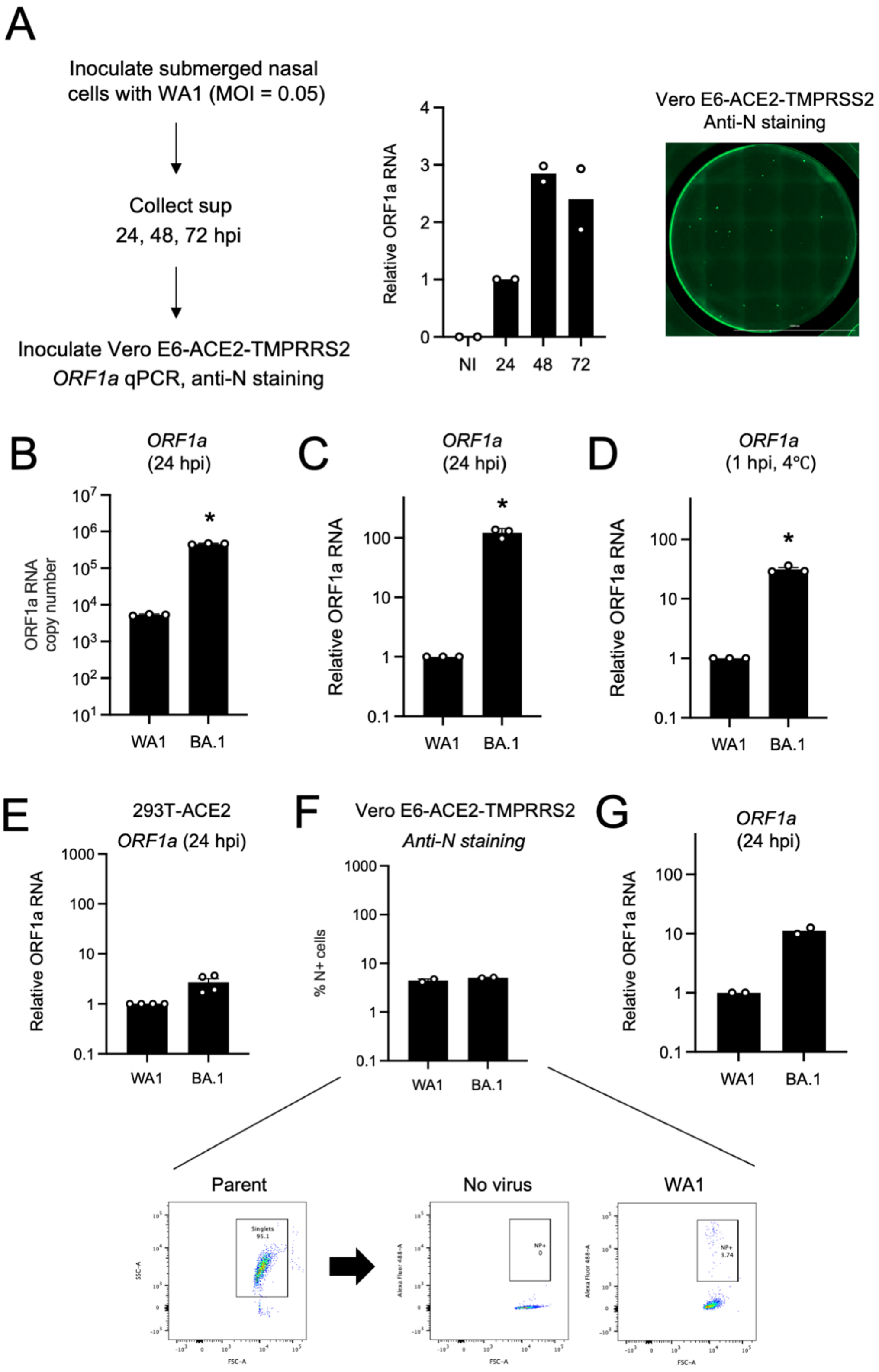

Supplemental Figure 2. Demonstration of productive infection of submerged nasal monolayers by SARS-CoV-2.

(A) Primary human nasal epithelial cells (pooled from 3 human donors) were cultured as undifferentiated, submerged monolayers and challenged with WA1 at an MOI of 0.05. Cell culture supernatants were collected at 24, 48, and 72 hours post-inoculation and added to Vero E6-ACE2-TMPRSS2 cells. 24 hours later, Vero E6-ACE2-TMPRSS2 cells were subjected to total RNA extraction and viral ORF1a RT-qPCR was performed. In addition, productive infection of Vero E6-ACE2-TMPRSS2 by the 24 hours post-inoculation supernatant was confirmed by anti-N immunofluorescence microscopy. (B) Primary human nasal epithelial cells (cells from three human donors, pooled) were inoculated with WA1 or BA.1 at an MOI of 0.05. Total cellular RNA was extracted and viral ORF1a was quantified by RT-qPCR at 24 hours post-inoculation. Absolute ORF1a RNA copy numbers were calculated by comparison to an ORF1a standard curve. (C) Relative viral ORF1a RNA abundance compared to actin was determined by the $2^{(-\Delta\Delta CT)}$ method. ORF1a abundance of WA1 was set to 1. (D) Primary human nasal epithelial cells (cells from three human donors, pooled) were inoculated with WA1 or BA.1 at an MOI of 0.05 on ice. At 1 hour post-inoculation, total cellular RNA was extracted and viral ORF1a was quantified by RT-qPCR to measure virus adherence to cells. Relative viral ORF1a RNA abundance compared to actin was determined by the $2^{(-\Delta\Delta CT)}$ method. ORF1a abundance of WA1 was set to 1. (E) HEK293T-ACE2 cells were inoculated with WA1 or BA.1 at an MOI of 0.05. 24 hours post-inoculation, total cellular RNA was extracted and viral ORF1a was quantified by RT-qPCR. Relative viral ORF1a RNA abundance compared to actin was determined by the $2^{(-\Delta\Delta CT)}$ method. ORF1a abundance of WA1 was set to 1. (F) Vero E6-ACE2-TMPRSS2 cells were inoculated with WA1 or BA.1 at an MOI of 0.05. 24 hours post-inoculation, cells were fixed, stained with anti-N antibody, and infection was scored by flow cytometry. A representative flow gating strategy is shown. (G) Primary human nasal epithelial cells (cells from three human donors, pooled) were inoculated with WA1 or BA.1 (5×10^{7} copies of absolute ORF1a RNA used as input). 24 hours post-inoculation, total cellular RNA was extracted and viral ORF1a was quantified by RT-qPCR. Relative viral ORF1a RNA abundance compared to actin was determined by the $2^{(-\Delta\Delta CT)}$ method. ORF1a abundance of WA1 was set to 1. All results are represented as means plus standard error from three independent infections (symbols represent biological replicates). Statistically significant differences (* $P < 0.05$) between the indicated condition of BA.1 and the corresponding condition of WA.1 were determined by student's unpaired two-tailed t test (actual *p* values: (B) 0.0001; (C) 0.0006; (D) 0.0002). Refer to Supplemental Dataset 1 for non-normalized data. Source data are found in a Source Data file.

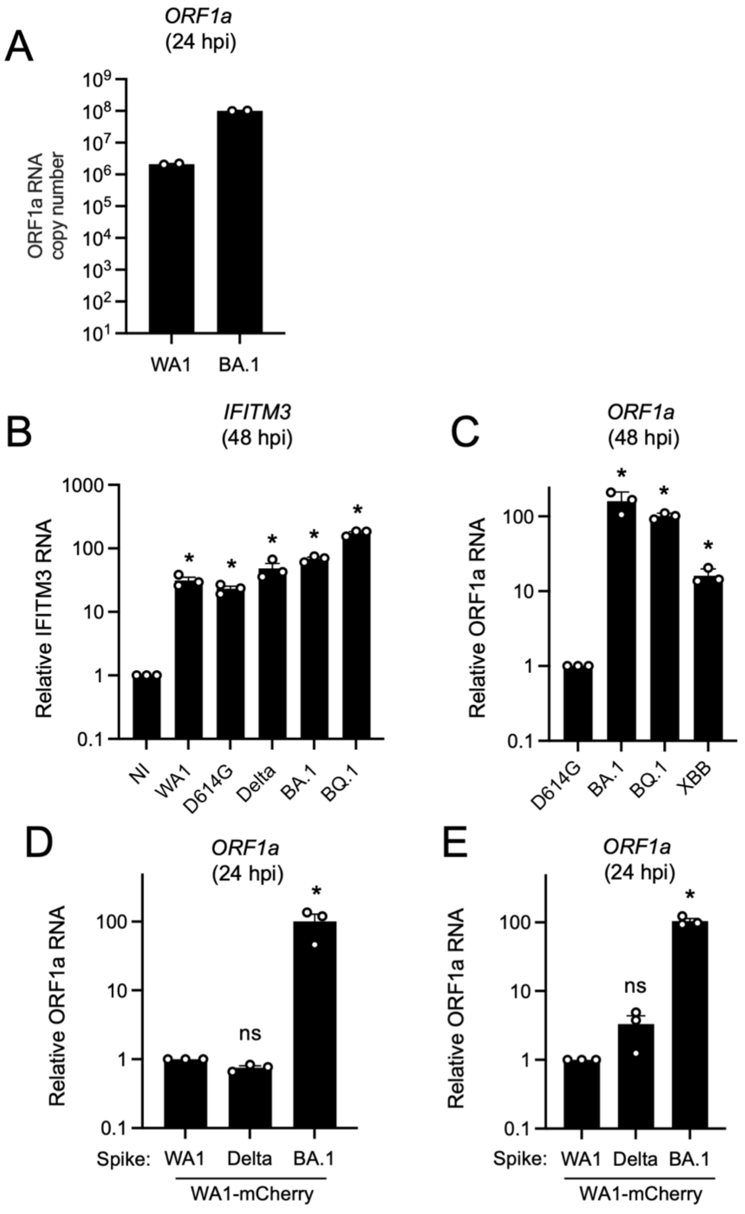

Supplemental Figure 3. Comparisons of infection rates achieved by SARS-CoV-2 variants in nasal ALI.

(A) Primary human nasal epithelial cells (pooled from 14 human donors) were cultured at the air-liquid interface and inoculated with 10000 plaque forming units of WA1 or BA.1. Total cellular RNA was extracted and viral ORF1a was quantified by RT-qPCR at 24 hours post-inoculation. Absolute ORF1a RNA copy numbers were calculated by comparison to an ORF1a standard curve. (B) Primary human nasal epithelial cells (pooled from 14 human donors) were cultured at the air-liquid interface and inoculated with 10000 plaque forming units of WA1, D614G, Delta, BA.1, or BQ.1. RT-qPCR of cellular *IFITM3* was performed at 48 hours post inoculation. Relative *IFITM3* transcript abundance was compared to actin using the $2^{(-\Delta\Delta CT)}$ method. *IFITM3* abundance in non-inoculated (NI) cells was set to 1. Statistically significant differences (* $P < 0.05$) between

the indicated condition and the NI condition were determined by one-way ANOVA adjusted for multiple comparisons (exact *p* values from left to right: 0.0173, 0.0310, 0.00236, 0.0006, 0.0001). (C) Primary human nasal epithelial cells (pooled from 14 human donors) were cultured at the air-liquid interface and inoculated with 10000 plaque forming units of D614G, BA.1, BQ.1, or XBB. At 48 hours post-inoculation, total cellular RNA was extracted and viral ORF1a was quantified by RT-qPCR. Relative ORF1a abundance was determined by comparing to actin using the 2^(-ΔΔCT) method. ORF1a abundance in D614G was set to 1. Statistically significant differences (* $P < 0.05$) between the indicated condition and D614G were determined by one-way ANOVA adjusted for multiple comparisons (exact *p* values from left to right: 0.0004, 0.0074, 0.0184). (D) 10000 plaque forming units of recombinant WA.1 encoding mCherry and Spike protein from WA1, Delta, or BA.1 (WA1 (WA1 Spike), WA1 (Delta Spike), and WA1 (BA.1 Spike)) were used to inoculate primary human nasal epithelial cells (pooled from 14 human donors) cultured at the air-liquid interface. At 48 hours post-inoculation, total cellular RNA was extracted and viral ORF1a was quantified by RT-qPCR. Relative ORF1a abundance was determined by comparing to actin using the 2^(-ΔΔCT) method. Statistically significant differences (* $P < 0.05$) between the indicated condition and WA1 (WA1 Spike) were determined by one-way ANOVA adjusted for multiple comparisons (exact *p* values from left to right: 0.99, 0.0081). (E) As in (D), except that 5x10^7 copies of absolute ORF1a RNA were used as input. At 48 hours post-inoculation, infection was measured by viral ORF1a RT-qPCR. Relative ORF1a abundance was determined by comparing to actin using the 2^(-ΔΔCT) method. Statistically significant differences (* $P < 0.05$) between the indicated condition and NI were determined by one-way ANOVA adjusted for multiple comparisons (exact *p* values from left to right: 0.588, 0.0001). All results are represented as means plus standard error from three independent infections (symbols represent biological replicates). Refer to Supplemental Dataset 1 for non-normalized data. Source data are found in a Source Data file.

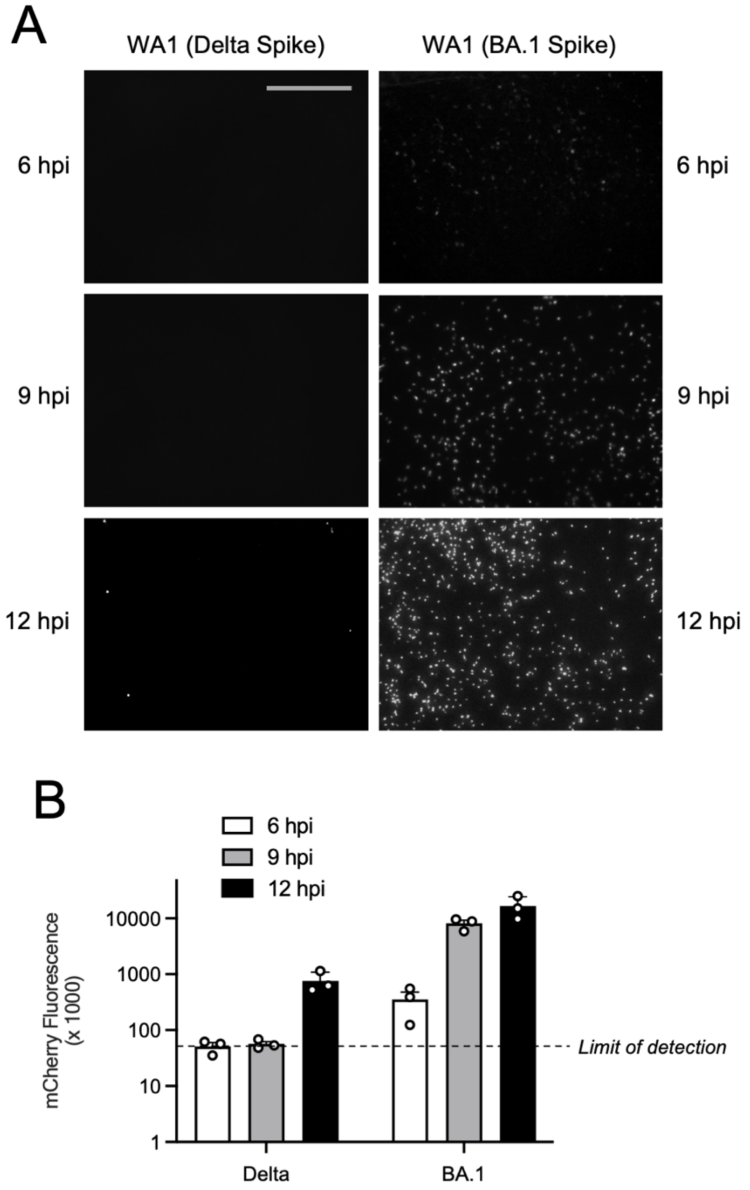

Supplemental Figure 4. Examination of recombinant virus infection and spread in nasal ALI at early timepoints.

(A) 10000 plaque forming units of recombinant WA.1 encoding mCherry and Spike protein from Delta or BA.1 (WA1-mCherry (Delta Spike), and WA1-mCherry (BA.1 Spike)) were used to inoculate primary human nasal epithelial cells (pooled from 14 human donors) cultured at the air-liquid interface. At 6, 9, and 12 hours post-inoculation, infection was measured by high-content imaging of mCherry fluorescence in living tissue. Scale bar = 300 microns. Note: mCherry fluorescence of WA1-mCherry (Delta Spike) was not apparent before 12 hours post-inoculation. (B) Quantification of mCherry fluorescence from each condition from three independent experiments. Results are represented as means plus standard error (symbols represent biological replicates). The limit of detection represents the mCherry fluorescence reading from non-inoculated tissue. Source data are found in a Source Data file.

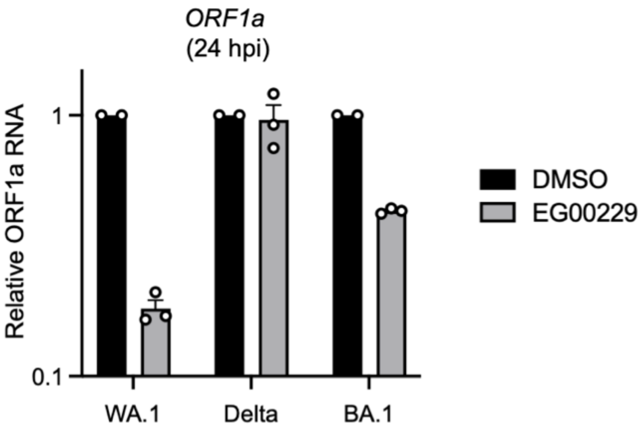

Supplemental Figure 5. The effect of neuropilin-1 inhibitor EG00229 on infection of nasal ALI by WA.1, Delta, and BA.1.

Primary human nasal epithelial cells (pooled from 14 human donors) were cultured at the air-liquid interface, pre-treated with 100 µM EG00229 or DMSO for two hours, and inoculated with 10000 plaque forming units of WA1, Delta, or BA.1. Viral ORF1a levels were measured by RT-qPCR at 24 hours post-inoculation. ORF1a RNA levels in the DMSO-treated condition for each virus were set to 1. Results are represented as means plus standard error from one infection (symbols represent three RT-qPCR runs). Refer to Supplemental Dataset 1 for non-normalized data. Source data are found in a Source Data file.

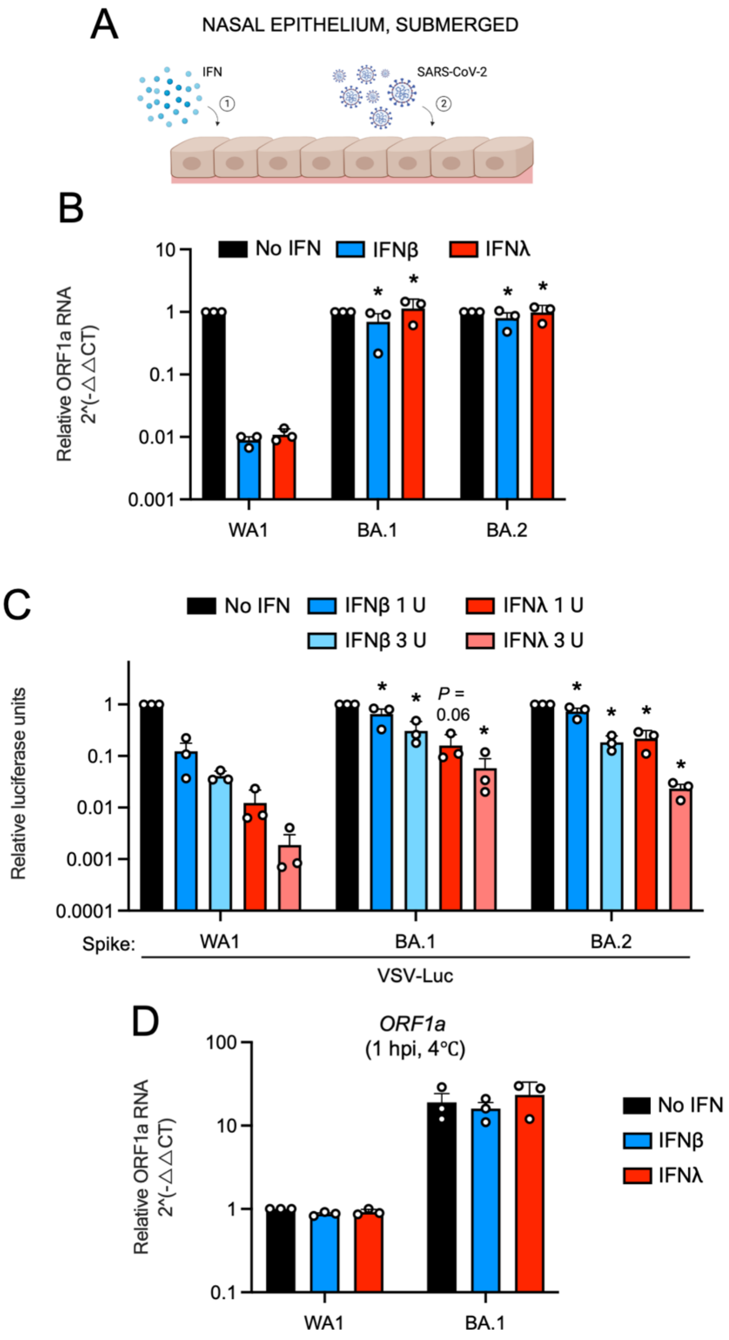

Supplemental Figure 6. Sensitivity of WA.1, BA.1, and BA.2 to type-I and type-III interferon pre-treatment in submerged nasal monolayers.

(A) Primary human nasal epithelial cells (pooled from 3 human donors) were cultured as undifferentiated, submerged monolayers, treated with IFN-beta or IFN-lambda for 18 hours, and challenged with SARS-CoV-2. Cartoon made with Biorender.com. (B) Cells were pre-treated with 2 units of IFN-beta or 2 units of FN-lambda for 18 hours, and inoculated with WA1, BA.1, or BA.2 at an MOI of 0.05. Total RNA was extracted from cells at 24 hours post inoculation, and

ORF1a levels were measured by RT-qPCR. Relative ORF1a abundance was determined by comparing to actin using the 2^(-ΔΔCT) method. For each virus, ORF1a levels in the absence of IFN were set to 1. (C) Primary human nasal epithelial cells (pooled from 3 human donors) were pre-treated with the indicated amounts of IFN-beta or IFN-lambda for 18 hours and challenged with VSV-based pseudovirus decorated with Spike from WA1, BA.1, or BA.2. At 24 hours post inoculation, luciferase activity was measured from lysed cells. Luciferase activity of WA1, BA.1, and BA.2 pseudoviruses in the absence of IFN were set to 1. (D) Primary human nasal epithelial cells (pooled from 3 human donors) were pre-treated with 2 units of IFN-beta or 5 ng/mL IFN-lambda for 18 hours and inoculated with WA1 or BA.1 at an MOI of 0.05 on ice. Total RNA was extracted from cells at 1 hour post inoculation, and ORF1a levels were measured by RT-qPCR. Relative ORF1a abundance was determined by comparing to actin using the 2^(-ΔΔCT) method. ORF1a levels of WA.1 in the absence of IFN were set to 1. All results are represented as means plus standard error from three independent infections (symbols represent biological replicates). Statistically significant differences (* $P < 0.05$) between the indicated condition and the corresponding WA1 condition were determined by one-way ANOVA adjusted for multiple comparisons (exact p values from left to right: (B) 0.0487, 0.0084, 0.0286, 0.0162; (C) 0.0343, 0.0435, 0.0624, 0.0392, 0.0197, 0.0171, 0.0205, 0.0128). Refer to Supplemental Dataset 1 for non-normalized data. Source data are found in a Source Data file.

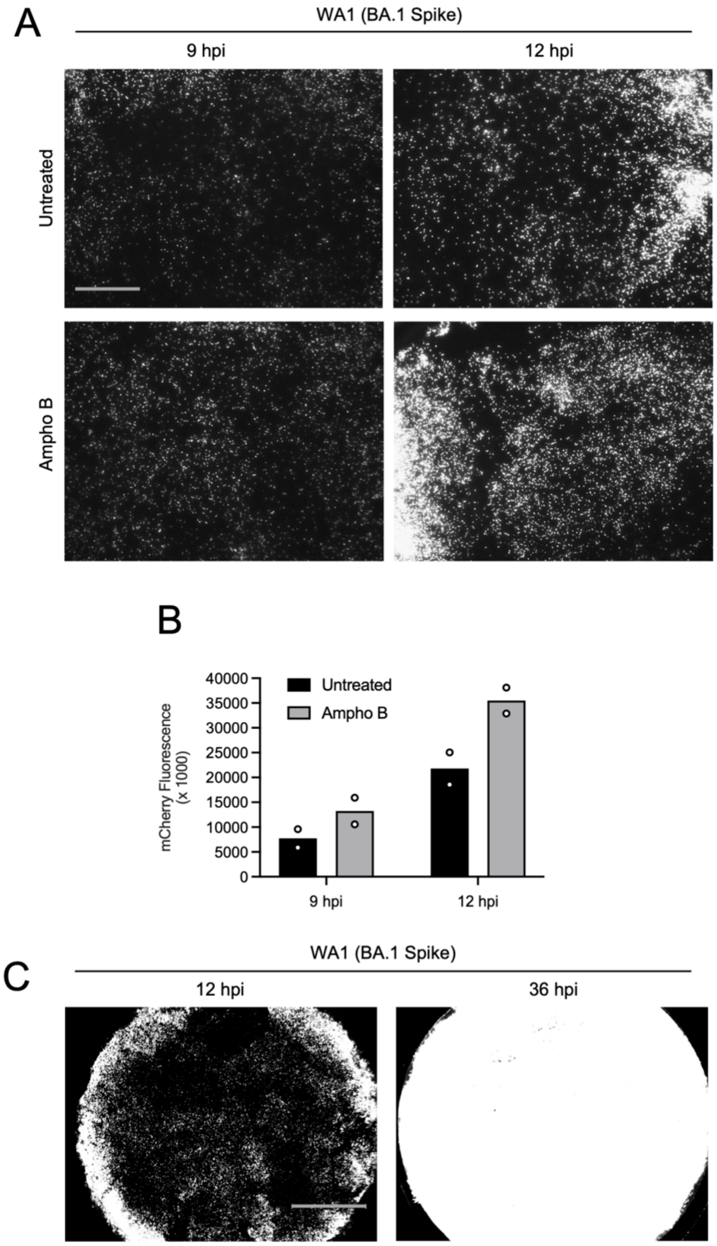

Supplemental Figure 7. Examination of the impact of Amphotericin B on spread of WA1-mCherry (BA.1 Spike) in nasal ALI.

(A) Primary human nasal epithelial cells (pooled from 14 human donors) were cultured at the air-liquid interface, pre-treated with 1 μM Amphotericin B for two hours or untreated, and inoculated with 10000 plaque forming units of WA1-mCherry (BA.1 Spike). mCherry fluorescence was measured by high-content imaging of living tissue at 9 and 12 hours post-inoculation. (B) Quantification of mCherry fluorescence in each condition from two independent experiments. (C) Tissue was fixed at 12 hours or 36 hours post-inoculation and mCherry fluorescence was measured by high-content imaging. Scale bar = 300 μm. Ampho B; amphotericin B. Source data are found in a Source Data file.

Supplemental Table 1. Titers of all virus stocks used in this study.

Strain	ORF1a RNA / 250 uL	FFU / 250 uL	ORF1a RNA in inoculant (equal FFU)
WA1	83,147,605	1,250,000	3,325,904
D614G	79,512,943	2,500,000	1,590,259
Delta	75,535,028	2,500,000	1,510,701
BA.1	104,287,953	250,000	20,857,591
BA.2	221,735,255	500,000	31,042,935
BQ.1	61,771,218	500,000	6,177,122
XBB	299,633,248	250,000	29,963,325
WA1-mCherry	505,205,925	85,000	59,876,258
WA1-mCherry (Delta S)	561,724,500	125,000	52,655,380
WA1-mCherry (BA.1 S)	105,626,354	50,000	26,825,741

Virus titers were determined by ORF1a RT-qPCR and focus-forming units assay in Vero E6-ACE2-TMPRSS2 cells. Results are displayed as ORF1a copy number per 250 uL or focus-forming units per 250 uL, respectively.
