# Supplementary material for: Omicron Spike confers enhanced infectivity and interferon resistance to SARS-CoV-2 in human nasal tissue
Source: Nat Commun. 2024 Jan 30;15:889. doi: 10.1038/s41467-024-45075-8 (PMC10828397; doi:10.1038/s41467-024-45075-8)
Supplement: Supplementary file 3 — Reporting Summary [file 41467_2024_45075_MOESM3_ESM.pdf]

nature portfolio

Corresponding author(s): Compton

Last updated by author(s): Dec 17, 2023

Reporting Summary

Nature Portfolio wishes to improve the reproducibility of the work that we publish. This form provides structure for consistency and transparency in reporting. For further information on Nature Portfolio policies, see our Editorial Policies and the Editorial Policy Checklist.

Statistics

For all statistical analyses, confirm that the following items are present in the figure legend, table legend, main text, or Methods section.

n/a	Confirmed	
☐	☒	The exact sample size (*n*) for each experimental group/condition, given as a discrete number and unit of measurement
☐	☒	A statement on whether measurements were taken from distinct samples or whether the same sample was measured repeatedly
☐	☒	The statistical test(s) used AND whether they are one- or two-sided<br>*Only common tests should be described solely by name; describe more complex techniques in the Methods section.*
☒	☐	A description of all covariates tested
☐	☒	A description of any assumptions or corrections, such as tests of normality and adjustment for multiple comparisons
☐	☒	A full description of the statistical parameters including central tendency (e.g. means) or other basic estimates (e.g. regression coefficient) AND variation (e.g. standard deviation) or associated estimates of uncertainty (e.g. confidence intervals)
☐	☒	For null hypothesis testing, the test statistic (e.g. *F*, *t*, *r*) with confidence intervals, effect sizes, degrees of freedom and *P* value noted<br>*Give P values as exact values whenever suitable.*
☒	☐	For Bayesian analysis, information on the choice of priors and Markov chain Monte Carlo settings
☒	☐	For hierarchical and complex designs, identification of the appropriate level for tests and full reporting of outcomes
☒	☐	Estimates of effect sizes (e.g. Cohen's *d*, Pearson's *r*), indicating how they were calculated

Our web collection on statistics for biologists contains articles on many of the points above.

Software and code

Policy information about availability of computer code

Data collection	*Provide a description of all commercial, open source and custom code used to collect the data in this study, specifying the version used OR state that no software was used.*
Data analysis	Graphpad Prism versions 10.1.0

For manuscripts utilizing custom algorithms or software that are central to the research but not yet described in published literature, software must be made available to editors and reviewers. We strongly encourage code deposition in a community repository (e.g. GitHub). See the Nature Portfolio guidelines for submitting code & software for further information.

Data

Policy information about availability of data

All manuscripts must include a data availability statement. This statement should provide the following information, where applicable:

- Accession codes, unique identifiers, or web links for publicly available datasets
- A description of any restrictions on data availability
- For clinical datasets or third party data, please ensure that the statement adheres to our policy

All data from this study is provided within the Figures, Supplemental Information, and the Source Data file.

Research involving human participants, their data, or biological material

Policy information about studies with human participants or human data. See also policy information about sex, gender (identity/presentation), and sexual orientation and race, ethnicity and racism.

Reporting on sex and gender	N/A
Reporting on race, ethnicity, or other socially relevant groupings	N/A
Population characteristics	N/A
Recruitment	N/A
Ethics oversight	N/A

Note that full information on the approval of the study protocol must also be provided in the manuscript.

Field-specific reporting

Please select the one below that is the best fit for your research. If you are not sure, read the appropriate sections before making your selection.

☒ Life sciences ☐ Behavioural & social sciences ☐ Ecological, evolutionary & environmental sciences

For a reference copy of the document with all sections, see nature.com/documents/nr-reporting-summary-flat.pdf

Life sciences study design

All studies must disclose on these points even when the disclosure is negative.

Sample size	Most experiments were performed three independent times (biological replicates) and symbols are to indicate each replicate. Sample size calculations were not performed. Sample sizes were chosen based on primary human tissue availability. Our sample sizes are sufficient because they involve the pooling of tissues from 3 to 14 human donors.
Data exclusions	There are no data exclusions.
Replication	All experiments were replicated at least 3 times and all attempts at replication were successful.
Randomization	N/A. We received human tissue that was already pooled from 3 to 14 human donors, whose identify was unknown to us, and so randomization was not applicable.
Blinding	N/A. We received human tissue that was already pooled from 3 to 14 human donors, whose identify was unknown to us, and so randomization was not applicable.

Reporting for specific materials, systems and methods

We require information from authors about some types of materials, experimental systems and methods used in many studies. Here, indicate whether each material, system or method listed is relevant to your study. If you are not sure if a list item applies to your research, read the appropriate section before selecting a response.

Materials & experimental systems

n/a	Involved in the study	
☐	☒	Antibodies
☐	☒	Eukaryotic cell lines
☒	☐	Palaeontology and archaeology
☒	☐	Animals and other organisms
☒	☐	Clinical data
☐	☒	Dual use research of concern
☒	☐	Plants

Methods

n/a	Involved in the study	
☒	☐	ChIP-seq
☐	☒	Flow cytometry
☒	☐	MRI-based neuroimaging

Antibodies

Antibodies used	Anti-nucleocapsid (Invitrogen MA1-7403), anti-ACE2 (abcam ab15348), anti-acetyl-a-tubulin (Cell Signaling, 5335). Secondary antibodies were DyLight 488 and DyLight 680.
Validation	Anti-nucleocapsid antibody (diluated 1:250) was validated by us by staining infected and non-infected tissue (as a control)-absence of signal was observed in the non-infected tissue. Anti-ACE2 antibody (diluted 1:250) was validated by us by staining lung cells (positive control) versus HEK293T cells (negative control)--no signal was observed in the HEK293T cells. Anti-acetyl-a-tubulin (diluted 1:250) was validated by us by staining ciliated nasal tissue (positive control) and non-ciliated HEK293T (negative control) cells. We observed little to no signal in non-cilated HEK293T cells. Secondary antibodies DyLight 488 and Dylight 680 (diluted 1:500) did not generate signal in the absence of primary antibodies.

Eukaryotic cell lines

Policy information about cell lines and Sex and Gender in Research

Cell line source(s)	Vero E6-ACE2-TMPRSS2 were obtained from BEI Resources (NR-54970). HEK293T-ACE2 were obtained from BEI Resources (NR-52511).
Authentication	Cell lines were authenticated by BEI Resources. The authors did not do an additional authentication.
Mycoplasma contamination	All cell lines were tested for mycoplasma contamination and they tested negative.
Commonly misidentified lines (See ICLAC register)	No commonly misidentified lines were used in this study.

Plants

Seed stocks	N/A
Novel plant genotypes	N/A
Authentication	N/A

Flow Cytometry

Plots

Confirm that:

- [x] The axis labels state the marker and fluorochrome used (e.g. CD4-FITC).
- [x] The axis scales are clearly visible. Include numbers along axes only for bottom left plot of group (a 'group' is an analysis of identical markers).
- [x] All plots are contour plots with outliers or pseudocolor plots.
- [x] A numerical value for number of cells or percentage (with statistics) is provided.

Methodology

Sample preparation	Vero E6-ACE2-TMPRSS2
Instrument	FACS Canto
Software	FlowJo
Cell population abundance	Immunostaining with anti-nucleocapsid antibody. Population abundance was performed by comparing to non-infected (negative control) cells.
Gating strategy	All events were plotted by SSC and FSC, then gated to exclude cell debris and cell clumps. The percentage of NP+ cells was measured from this subpopulation. Non-infected cells were used as a negative control to set the NP-positive gate.

- [x] Tick this box to confirm that a figure exemplifying the gating strategy is provided in the Supplementary Information.
