# Supplementary material for: Omicron Spike confers enhanced infectivity and interferon resistance to SARS-CoV-2 in human nasal tissue
Source: Nat Commun. 2024 Jan 30;15:889. doi: 10.1038/s41467-024-45075-8 (PMC10828397; doi:10.1038/s41467-024-45075-8)
Supplement: Supplementary file 4 — Description of Additional Supplementary files [file 41467_2024_45075_MOESM4_ESM.pdf]

Description of Additional Supplementary Items

File name: Supplementary Data 1
Description: Non-normalized figures
