# Supplementary material for: Omicron Spike confers enhanced infectivity and interferon resistance to SARS-CoV-2 in human nasal tissue
Source: Nat Commun. 2024 Jan 30;15:889. doi: 10.1038/s41467-024-45075-8 (PMC10828397; doi:10.1038/s41467-024-45075-8)

Non-normalized data from Figure 1

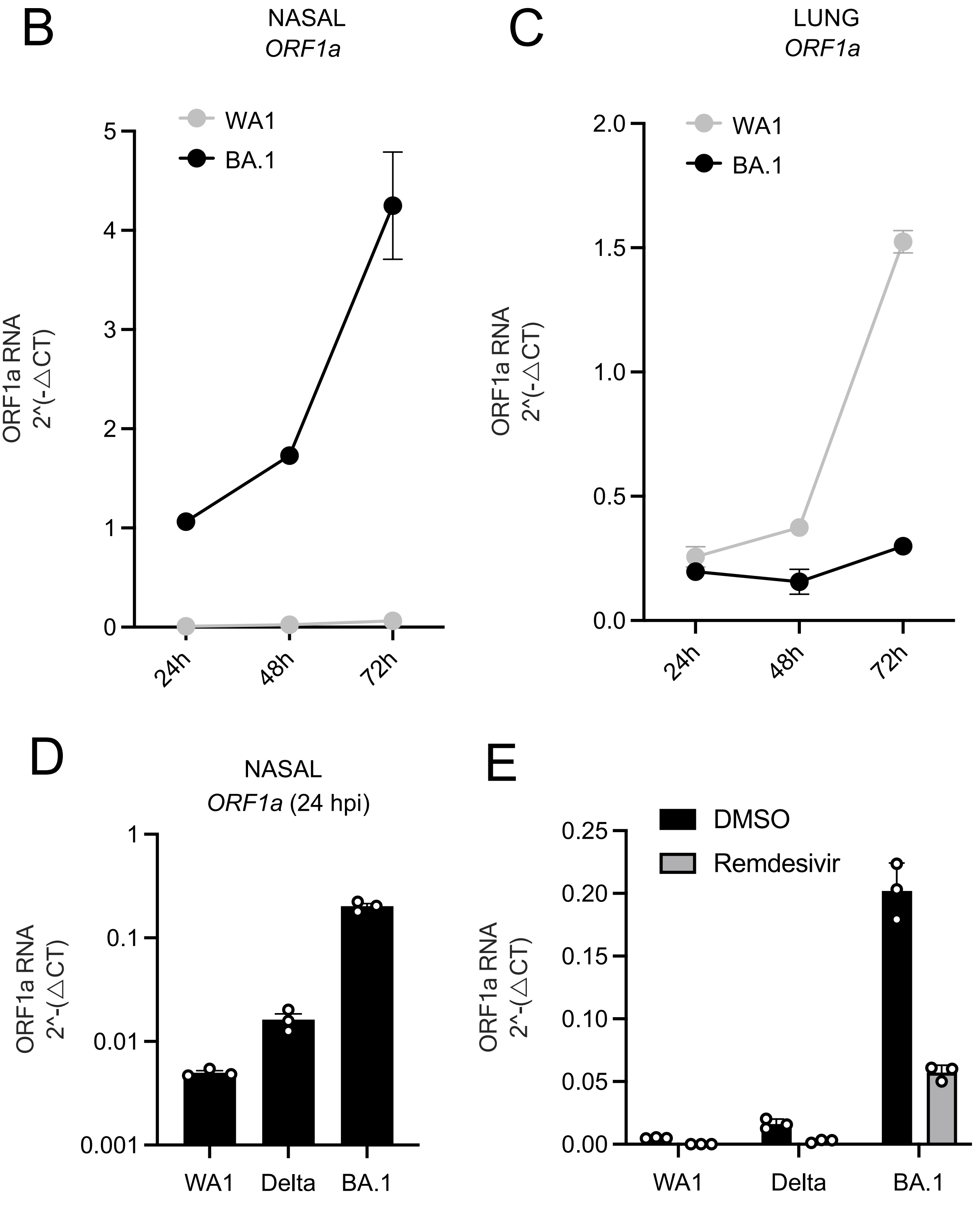

Non-normalized data from Figure 2

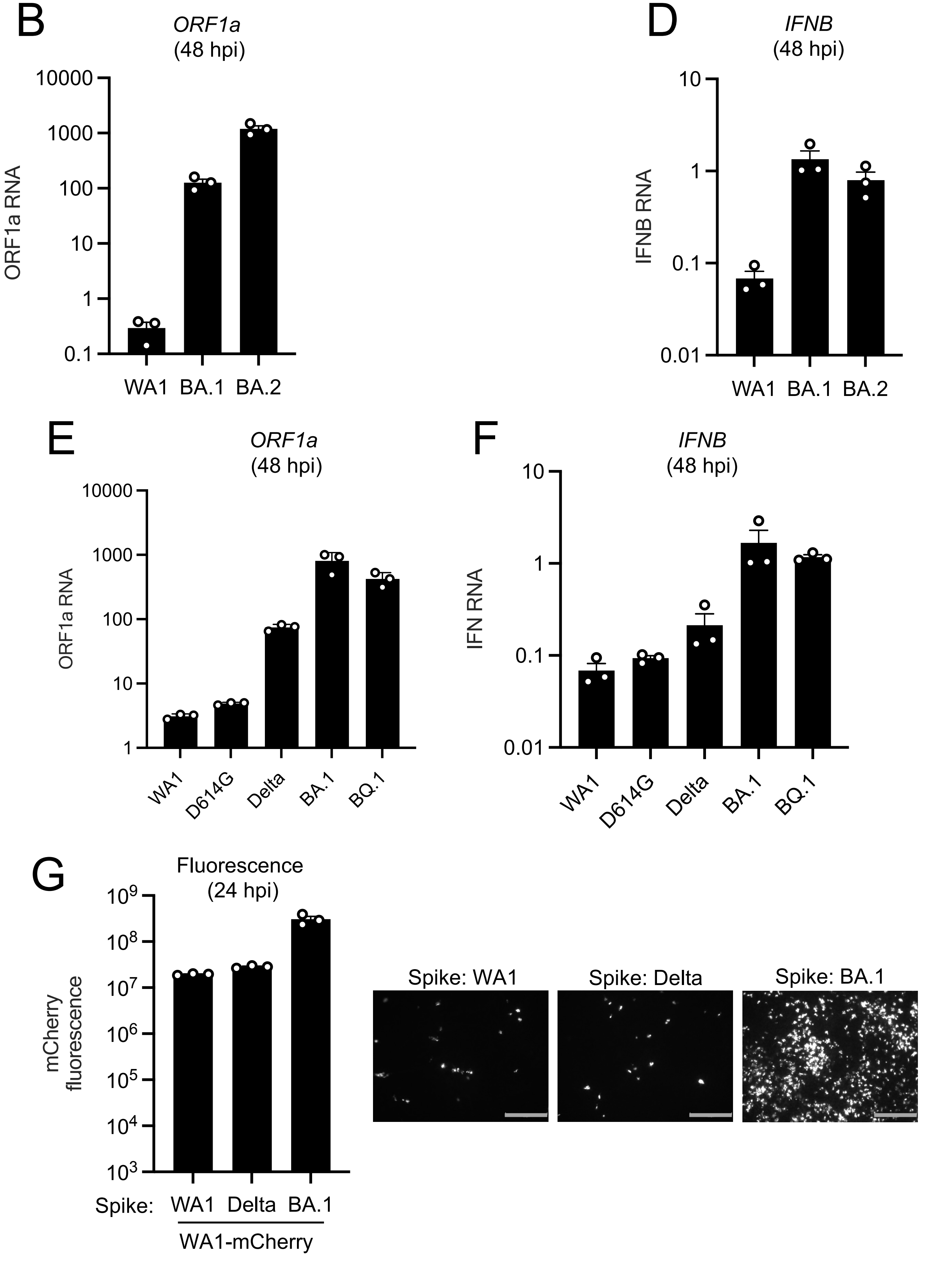

Non-normalized data from Figure 3

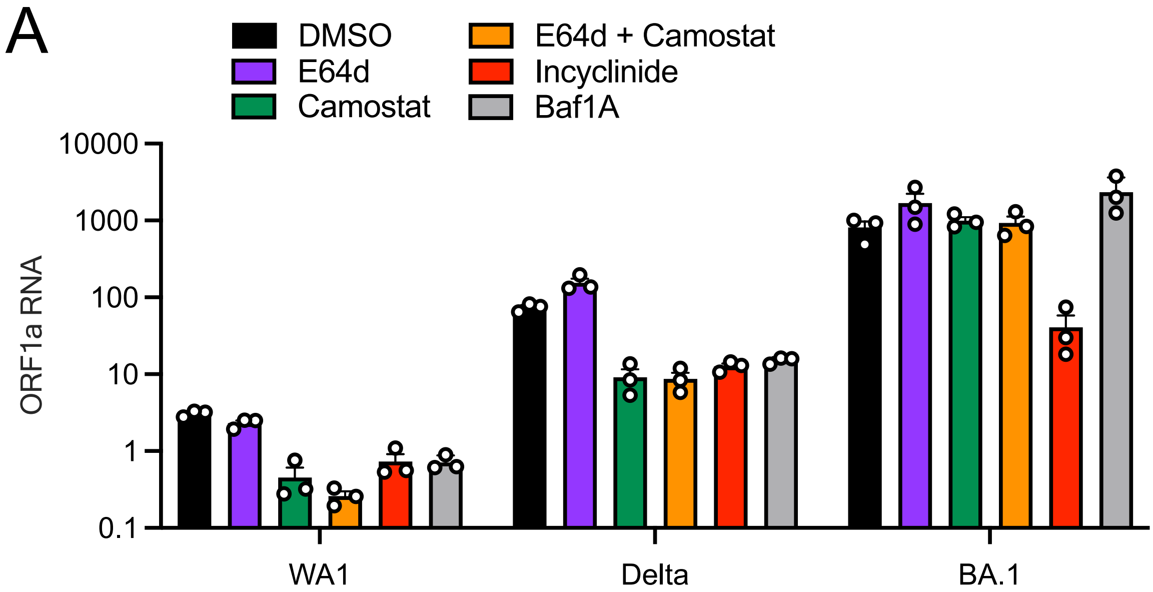

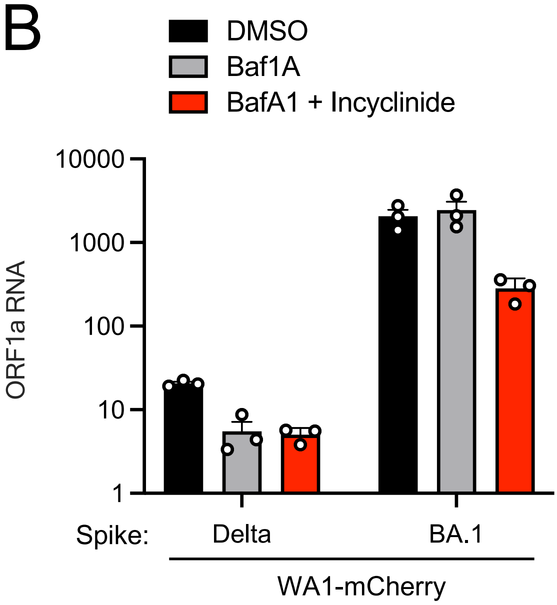

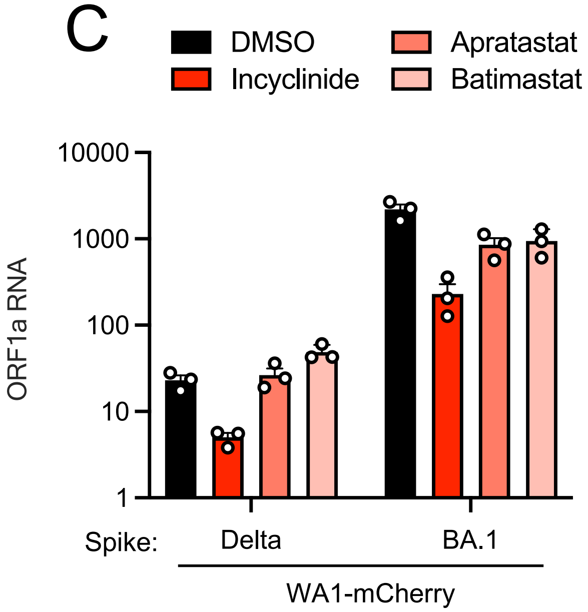

Non-normalized data from Figure 4

B

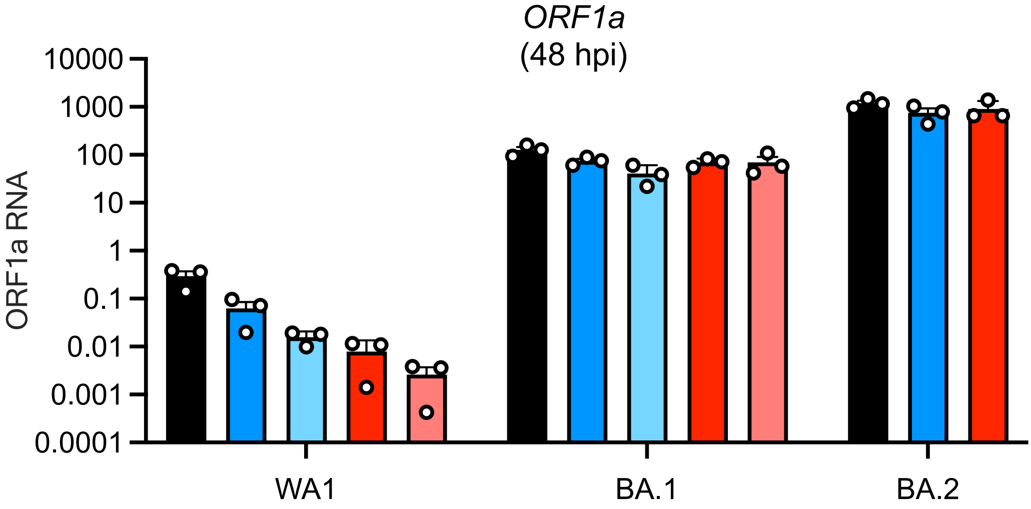

D

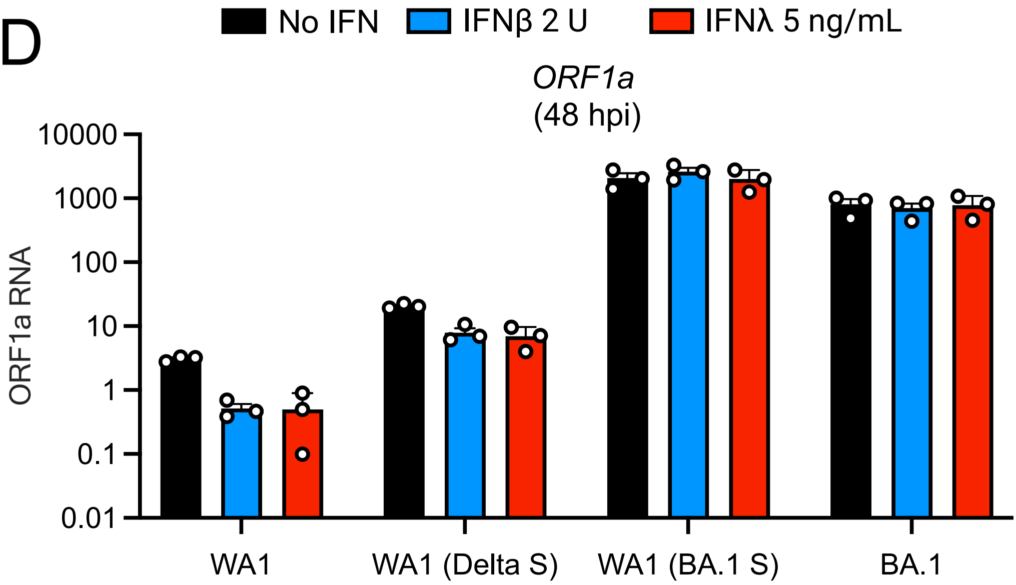

Non-normalized data from Supp. Figure 2

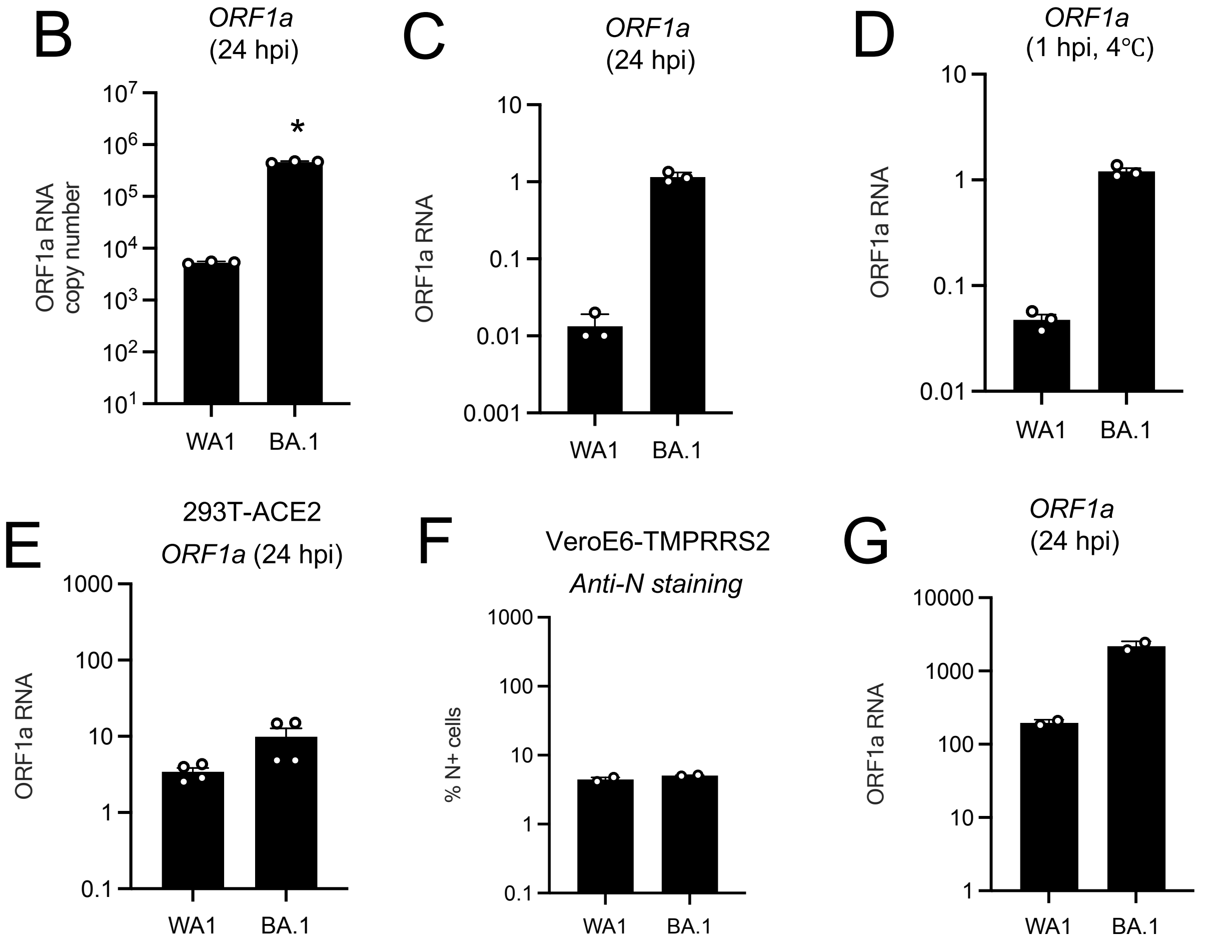

Non-normalized data from Supp. Figure 3

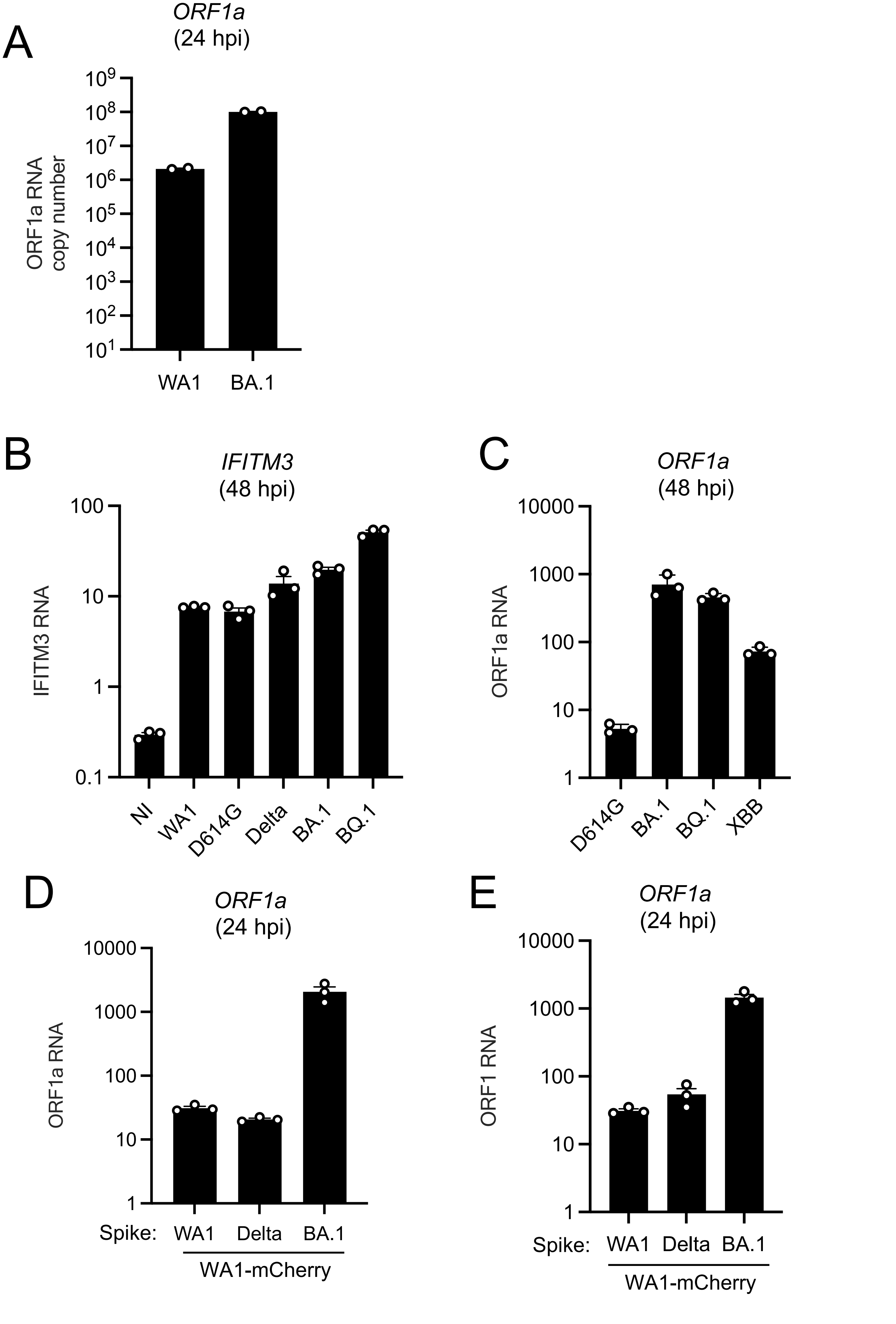

Non-normalized data from Supp. Figure 5

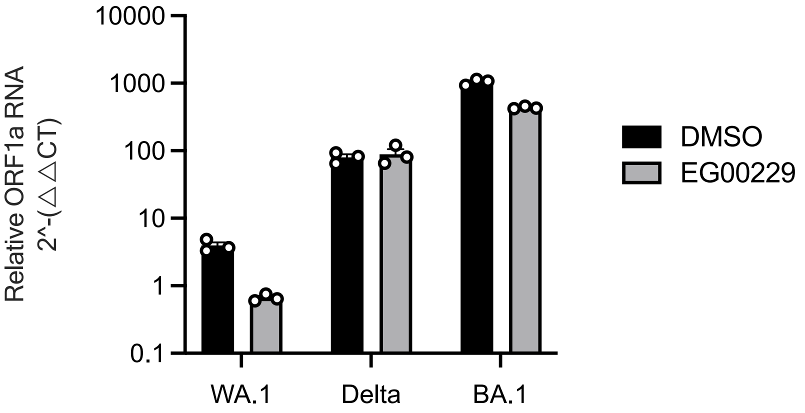

Non-normalized data from Supp. Figure 6

B

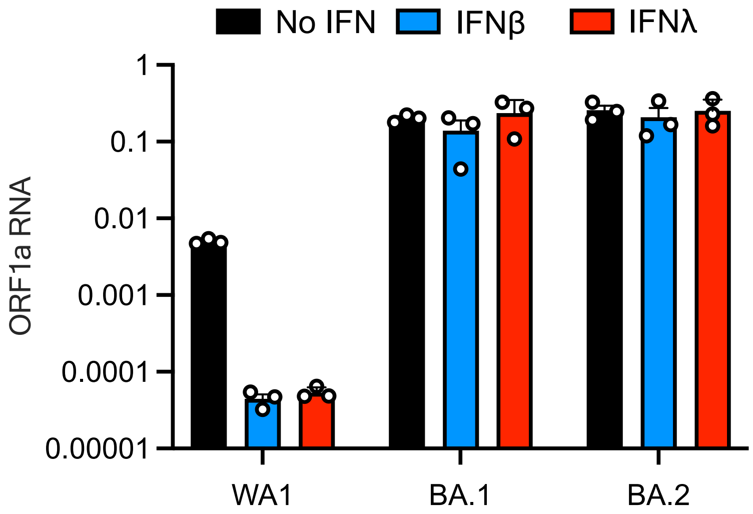

D

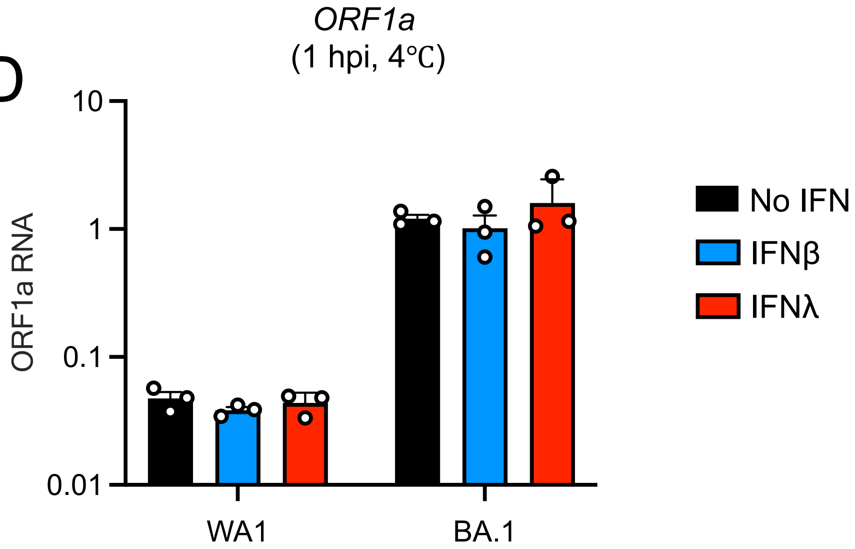

Supplement: Supplementary file 5 — Supporting Dataset 1 [file 41467_2024_45075_MOESM5_ESM.pdf]
